# Rosiglitazone-Mediated Activation of PPARγ Induces PlGF Expression in Trophoblast Cells

**DOI:** 10.1007/s43032-025-01868-w

**Published:** 2025-04-28

**Authors:** Pinki Nandi, Chidambra Halari, Mavis Lee, Elakkiya Prabaharan, Shahil Sarajideen, Dennis K. Lee, Sascha Drewlo

**Affiliations:** 1 Sunnybrook Research Institute, Toronto, ON Canada; 2 Department of Obstetrics & Gynaecology, University of Toronto, Toronto, ON Canada; 3 Department of Laboratory Medicine and Pathobiology, University of Toronto, Toronto, ON Canada

**Keywords:** Preeclampsia, PPARγ, Rosiglitazone, Placental growth factor, GCM1, HO-1

## Abstract

Preeclampsia (PE) is a hypertensive pregnancy disorder marked by impaired trophoblast invasion and placental vascular dysfunction, resulting in severe maternal and fetal complications. Placental growth factor (PlGF) is critical for proper placental angiogenesis and is transcriptionally regulated by glial cell missing-1 (GCM1), a downstream effector of peroxisome proliferator-activated receptor-gamma (PPARγ). Decreased PPARγ activity in PE may therefore contribute to diminished PlGF levels, worsening placental pathology. In this study, we investigated the mechanistic role of rosiglitazone, a PPARγ agonist, in rescuing PlGF expression under 1.5% oxygen/reoxygenation stress mimicking PE. Using JEG-3 trophoblast cells, we show that rosiglitazone enhances PPARγ nuclear translocation, leading to increased GCM1 and cyto-protective heme oxygenase-1 (HO-1) expression, and subsequent upregulation of PlGF production under both 21% oxygen and 1.5% oxygen/reoxygenation conditions. Pharmacologic inhibition of PPARγ with T0070907 or siRNA-mediated knockdown abrogated these effects, underscoring PPARγ’s essential role in maintaining GCM1-driven PlGF expression. Notably, rosiglitazone treatment rescued PlGF production in 1.5% oxygen/reoxygenation-stressed cells, highlighting a potential therapeutic strategy to mitigate placental dysfunction. These findings define the PPARγ-GCM1-PlGF axis as a mechanistic cornerstone of placental health and suggest that pharmacological activation of PPARγ may offer clinical benefit in improving pregnancy outcomes in PE.

**Supplementary Information:**

The online version contains supplementary material available at 10.1007/s43032-025-01868-w.

## Introduction

Preeclampsia (PE) is a hypertensive disorder of pregnancy believed to originate from abnormal placentation and results in both placental and systemic maternal complications. Globally, PE is a leading cause of maternal and fetal morbidity and mortality, affecting up to 8% of all pregnancies. Delayed diagnosis can result in severe postpartum complications that damage multiple maternal organ systems. Among the most severe complications are preterm delivery before 37 weeks and long-term cardiovascular disease risks for both the mother and child [1].

The underlying pathology of PE is hypothesized to involve abnormal trophoblast function, including reduced extravillous trophoblast (EVT) invasion and inadequate remodelling of the spiral arteries during the 1st trimester [2, 3]. Failure of the EVTs to remodel the spiral arteries might impair uteroplacental circulation [2], leading to placental hypoperfusion and a prolonged low oxygen state. This is thought to promote oxidative stress through the generation of reactive oxygen species and free radicals, further disrupting trophoblast function and normal villous development [4, 5]. One proposed consequence of abnormal placentation is the reduced placental secretion of proangiogenic proteins such as placental growth factor (PlGF) and increased expression of anti-angiogenic factors such as soluble fms-like tyrosine kinase 1 (sFlt1) into the maternal circulation. Imbalance of placental-derived proteins disrupts signaling pathways in endothelial cells, causing systemic endothelial dysfunction and hypertension [2, 6].

PlGF plays an important role in mediating vasculogenesis and angiogenesis during placentation. As a member of the vascular endothelial growth factor family, PlGF interacts with maternal endothelial cell receptors to facilitate systemic vasodilation, critical for managing the increased blood volume and cardiac output observed during pregnancy [7]. In normal pregnancy, levels of circulating PlGF in maternal blood increase consistently until the onset of the third trimester, indicating the progress of both uteroplacental circulation and placental growth [8]. In contrast, low circulating PlGF levels precede the onset of clinical symptoms in PE pregnancies [9] and a cutoff value of 100 pg/mL demonstrates high diagnostic accuracy for PE and placenta-mediated fetal growth restriction between 20 and 36 weeks [8]. Experimental animal models of PE have shown promise with supplemental PlGF treatment, suggesting it could potentially alleviate the disease and providing strong impetus for further research into PlGF as a target for pharmacological intervention [9].

The pathogenesis of PE is intricately linked to disrupted differentiation of cytotrophoblast into syncytiotrophoblasts and EVTs [10]. This disruption is associated with reduced expression of key regulatory proteins, including peroxisome proliferator-activated receptor gamma (PPARγ) and glial cell missing 1 (GCM1). PPARγ, a nuclear steroid receptor, is expressed in villous trophoblasts throughout pregnancy and in 1st trimester EVTs [11, 12]. GCM1, expressed by cytotrophoblasts and the syncytiotrophoblast layer, is upregulated by PPARγ via binding to PPARγ response elements in its promoter [13]. Previous studies showed that PPARγ and GCM1 expressions are significantly reduced in PE [14–16].

PlGF is predominantly expressed by trophoblast cells in the placenta and its expression is regulated by GCM1 [17, 18]. Both GCM1 and the protein syncytin, which facilitates syncytial fusion [19, 20] in the placenta, may promote the expression and secretion of PlGF by the syncytiotrophoblast into the maternal circulation [18, 21]. In prolonged low oxygen conditions characteristic of PE, a reduction in GCM1 expression occurs, which subsequently results in decreased PlGF expression in the villi [15].

Previous studies have suggested that pharmacological induction of PPARγ can restore placental dysfunction and improve the imbalance of placental-derived angiogenic and antiangiogenic factors [16]. Our group has previously shown that pharmacological induction of PPARγ via rosiglitazone, a PPARγ agonist, reduces sFlt1 expression in explanted human first trimester placental villi [22]. Rosiglitazone has demonstrated multiple beneficial effects in trophoblast cells, such as enhancing trophoblast survival under low oxygen conditions by improving mitochondrial function. Additionally, rosiglitazone restores heme oxygenase-1 (HO-1) levels in the preeclamptic placenta, a crucial vasodilator that is deficient in PE. HO-1 is a cytoprotective and proangiogenic molecule that may have a significant role in decreasing the risk of hypertension and endothelial dysfunction [23].

In this in vitro pilot study, we tested the hypothesis that rosiglitazone-mediated PPARγ activation upregulates PlGF expression and promotes trophoblast differentiation in a placental 1.5% oxygen/reoxygenation injury model. Using the JEG-3 choriocarcinoma cell line, we pharmacologically manipulated PPARγ activity with rosiglitazone (agonist) and T0070907 (antagonist). Furthermore, PPARγ expression was silenced using siRNA to determine its effect on basal PlGF levels. Our study provides foundational data to suggest that the PPARγ-GCM1-PlGF axis may be a potential therapeutic target for PE.

## Methods

### Cell Culture

The JEG-3 human choriocarcinoma cell line was cultured in Dulbecco’s Modified Eagle Medium (DMEM) growth medium complete with 10% fetal bovine serum (Thermo Fisher Scientific, Waltham, USA) and 1% antibiotic-antimycotic (Thermofisher Scientific). The cells were cultured in 21% oxygen conditions (21% O_2,_ 5% CO_2_ at 37 °C) and medium was changed every 2 to 3 days. The cells were passaged using trypsin-ethylenediaminetetraacetic acid (Wisent, Saint-Jean-Baptiste, Canada) then seeded at 300,000 cells per well in 6-well plates and grown for 42 h in the same 21% oxygen culture conditions.

### Cell Treatment and 1.5% Oxygen/Reoxygenation Conditions

To induce oxidative stress similar to that experienced in the PE placenta, JEG-3 cells were cultured according to a low oxygen/reoxygenation protocol adapted from methods previously published by our lab [5]. Briefly, JEG-3 cells were plated and incubated in 21% oxygen conditions with either 10 or 50 µM of rosiglitazone (Selleck Chemicals, Houston, USA), or with dimethyl sulfoxide (MilliporeSigma Canada, Oakville, Canada) vehicle control for 18 h. After replacing the media, cells were supplemented with rosiglitazone or vehicle control and placed in 1.5% oxygen conditions (1.5% O_2,_ 5% CO_2_ at 37 °C) for 6 h. The media was replenished with rosiglitazone and returned to 21% oxygen conditions for 18 h of reoxygenation.

### RNA Extraction & RT-qPCR

Cells were lysed in QIAzol lysis reagent (Qiagen, Germantown, USA), and total RNA was extracted using the manufacturer’s protocol. The extracted RNA was quantified and assessed using a Nanodrop 2000c spectrophotometer (Thermo Fisher Scientific). Using 1 µg of RNA, cDNA was generated with the High-Capacity cDNA Reverse Transcription Kit (Thermo Fisher Scientific). Rt-qPCR was performed at 14 µl total reaction volume containing 25 ng of template cDNA, 7 µl SYBR-green Master Mix (Applied Biosystems, Waltham, USA), and 286 nM of forward and reverse primers (See Table 1). Relative changes in gene expression were analyzed using the DDCt method as described in [22].

**Table 1:** Quantitative real-time PCR primer sequences

Gene Name	Gene Symbol	Sequence
b-actin	*Actb*	F: 5’-CAT CAT CAA CAT CTT GAG CC-3’ R: 5’-CAG ATA GCC AAG GAT GTG TG-3’
Glial cell missing 1	*Gcm1*	F: 5’-TGA ACA CAG CAC CTT CCT C-3’ R: 5’-CCA CTG TAA CTA CCA GGC AAT-3’
Glyceraldehyde-3-phosphate dehydrogenase	*Gapdh*	F: 5’-CAC ATC ACA GCT CCC CAC CA-3’ R: 5’-TGC ACA GGA GCC AAG AGT GAA-3’
Heme oxygenase 1	*Hmox1*	F: 5’-ACA CTC TGG AGA TGA CAC CT-3’ R: 5’-TTG TGT TCC TCT GTC AGC ATC-3’
Peroxisome proliferator activated gamma	*PPARγ*	F: 5’-GGG ATC AGC TCC GTG GAT CT-3’ R: 5’-GGG AAG AAA TAG AAG GCT TTC-3’
Placental growth factor	*Pgf*	F: 5’-CCG CCA GGA CAA ACC AGT AT-3’ R: 5’-ACT TTT GGT ACA TTG TGG CTT CAA-3’

### Protein Extraction & Immunoblotting

Protein was extracted from the JEG-3 cells using RIPA buffer (Thermo Fisher Scientific) containing protease and phosphatase inhibitor cocktails (MilliporeSigma Canada). Protein concentration was then quantified with a BCA protein assay kit (Thermo Fisher Scientific). Western blot analyses were performed for each sample set using 25 µg of protein that were denatured (5 min, 95 °C) in Laemmli sample buffer (Bio-Rad Laboratories, Hercules, USA) and separated with sodium dodecyl sulfate-polyacrylamide gel electrophoresis (SDS-PAGE), followed by a wet transfer onto a polyvinylidene difluoride (PVDF) membrane. The membranes were blocked with 5% non-fat dry milk in Tris-buffered saline containing 0.05% Tween-20, then incubated overnight at 4 °C with anti-GCM1 (1:1000; Aviva Biosystems, San Diego, USA), anti-HO1 (1:500; Cell Signaling Technology, Danvers, USA), or anti-PPARγ (1:1000; Cell Signaling Technology) primary antibodies. Horseradish peroxidase-conjugated secondary antibodies were added to the membranes for 2 h at room temperature (RT) and developed with Pierce™ ECL western blotting substrate (Thermo Fisher Scientific). Subsequently, signals were visualized on a ChemiDoc™ imaging system (Bio-Rad Laboratories) and Image Lab Version 5.1 software (Bio-Rad Laboratories). Protein levels were normalized to a housekeeping protein, β‐actin (1:5000; Cell Signaling Technology).

### Immunological Assay

To measure PlGF secretion, enzyme-linked immunosorbent assay (ELISA) was performed on JEG-3 cell culture media. The media was centrifuged at 1500 rpm for 10 min at 4 °C and the supernatant collected. Levels of PlGF were assessed according to the manufacturer’s instructions (R&D Systems, Minneapolis, USA) and optical density of the assay product was measured at 450 and 540 nm (for correction) using a Synergy H1 microplate reader (BioTek, Winooski, USA). A standard curve was generated to calculate the concentration of PlGF, and values were normalized to total number of cells plated or total protein concentration.

### siRNA-mediated Suppression of PPARγ and GCM1

To demonstrate the knockdown of PPARγ and GCM1, Silencer™ Select pre-designed siRNA assays (Thermo Fisher Scientific) were utilized and Silencer™ Select negative control No. 1 siRNA (Thermo Fisher Scientific) was used as negative control. Silencing experiments were performed according to the manufacturer’s instruction. Briefly, JEG-3 cells were seeded in a 6-well plate and grown to 50–60% confluency in antibiotic-free DMEM media. The next day, culture media was removed, washed with PBS, and cells were subsequently incubated with fresh antibiotic-free DMEM media. A solution of 50–75 pmol siRNAs were prepared using Opti-MEM™ I reduced serum medium (Thermo Fisher Scientific) and Lipofectamine™ RNAiMAX transfection reagent (Thermo Fisher Scientific), incubated at RT for 20 min and added dropwise to the cells. Transfection media was diluted after 5 h and replaced with complete DMEM media containing antibiotics after 24 h. Cells were cultured for another 24 h before protein extraction.

### Immunocytochemistry

To show PPARγ nuclear translocation upon rosiglitazone treatment, cells were plated on an 8-well chamber slide (SPL Life Science, Pocheon-si, Korea) and grown to 50% confluency before treatment. Following treatment, cells were washed 3 times with PBS and fixed with 4% paraformaldehyde (PFA) for 30 min at RT. After fixation, PFA was removed, and cells were washed 3 times with PBS, then permeabilized with 0.1% Tween-20 for 10 minutes. Cells were then blocked with 5% bovine serum albumin in PBS for 1 h at RT and incubated with anti-PPARγ primary antibody (1:250, Thermo Fisher Scientific) for 2 h. Cells were washed 3 times with PBS and incubated with Alexa-fluor 488 anti-rabbit secondary antibody (Thermo Fisher Scientific, 4 ug/ml concentration) for 45 min. Excess secondary antibody was removed by washing 3 times with PBS washes. Excess PBS was wiped carefully from the slides with Kimwipes. ProLong™ Diamond antifade mountant with 4’,6-diamidino-2-phenylindole (DAPI, Thermo Fisher Scientific) was added and coverslips were mounted. Slides were left to dry for 24 h before imaging by confocal microscopy (Nikon A1 laser scanning confocal microscope).

### Statistical Analysis

All statistical analysis was performed with GraphPad Prism 10.4.1 software. mRNA and protein expression values were normalized to respective housekeeping genes or protein. Relative mRNA and protein expression values from cells treated with either vehicle, rosiglitazone, T0070709, or PPARγ-siRNA and scramble siRNA were first normalized to respective housekeeping genes or protein. Subsequently, the relative expression values for each biological replicate set were normalized to respective vehicle control or scramble siRNA control. ELISA data was adjusted based on cell number or total protein concentration. Groups were analyzed by student’s t-test or one-way ANOVA. *P* < 0.05 is considered significant and indicated with (*) on each graph. Bar plot data were represented as mean ± SEM.

## Results

### Rosiglitazone Induced PPARγ Activity in Trophoblast Cells

**Fig. 1:**
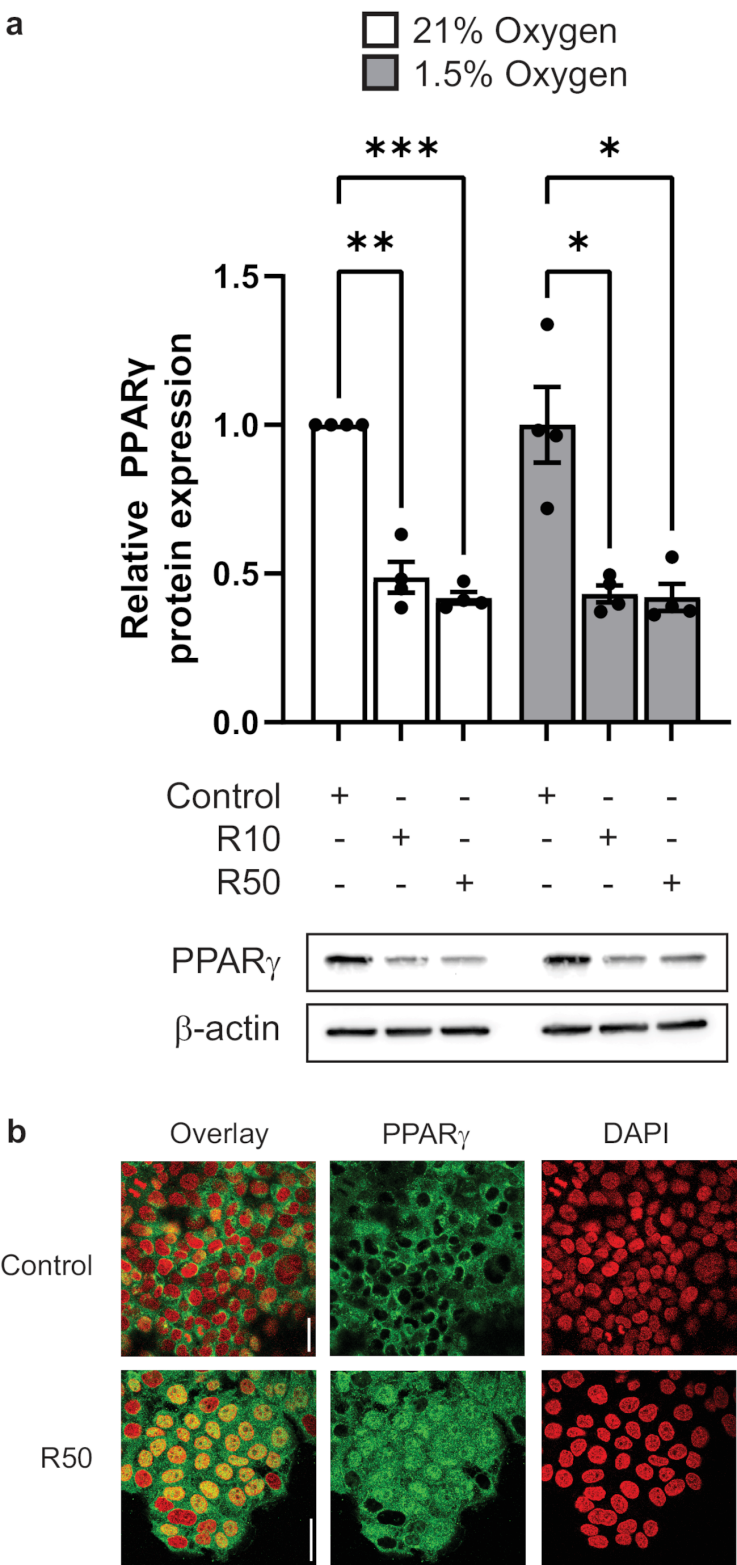
Rosiglitazone induces nuclear translocation and down-regulation of PPARγ. JEG-3 cells exposed to 21% O_2_ or 1.5% O_2_ (with reoxygenation at 21% O_2_) conditions in the presence of 10 (R10) or 50 (R50) µM rosiglitazone. (**a**) Western blot analyses of PPARγ levels normalized to β-actin by one-way ANOVA with post hoc tests to determine significant differences between groups; *P ≤ 0.05, **P ≤ 0.01, ***P ≤ 0.001 (*n* = 3). (**b**) Representative confocal microscopy images of PPARγ localization (green) under basal conditions and in the presence of 50 µM rosiglitazone. DAPI staining is shown in red and nuclear colocalization in yellow; bar = 50 μm

To demonstrate rosiglitazone mediated PPARγ activation, JEG-3 cells were treated with either 10 or 50 µM doses of rosiglitazone under 21% oxygen or 1.5% oxygen/reoxygenation conditions. As expected, PPARγ protein expression was reduced upon rosiglitazone treatment under both 21% oxygen and 1.5% oxygen/reoxygenation conditions, indicating its activation (Fig. 1a). Rosiglitazone-mediated PPARγ activation was further confirmed by PPARγ nuclear translocation under 21% oxygen conditions (Fig. 1b).

### Rosiglitazone-Mediated Activation of PPARγ Signaling Induced the Expression and Secretion of PlGF

**Fig. 2:**
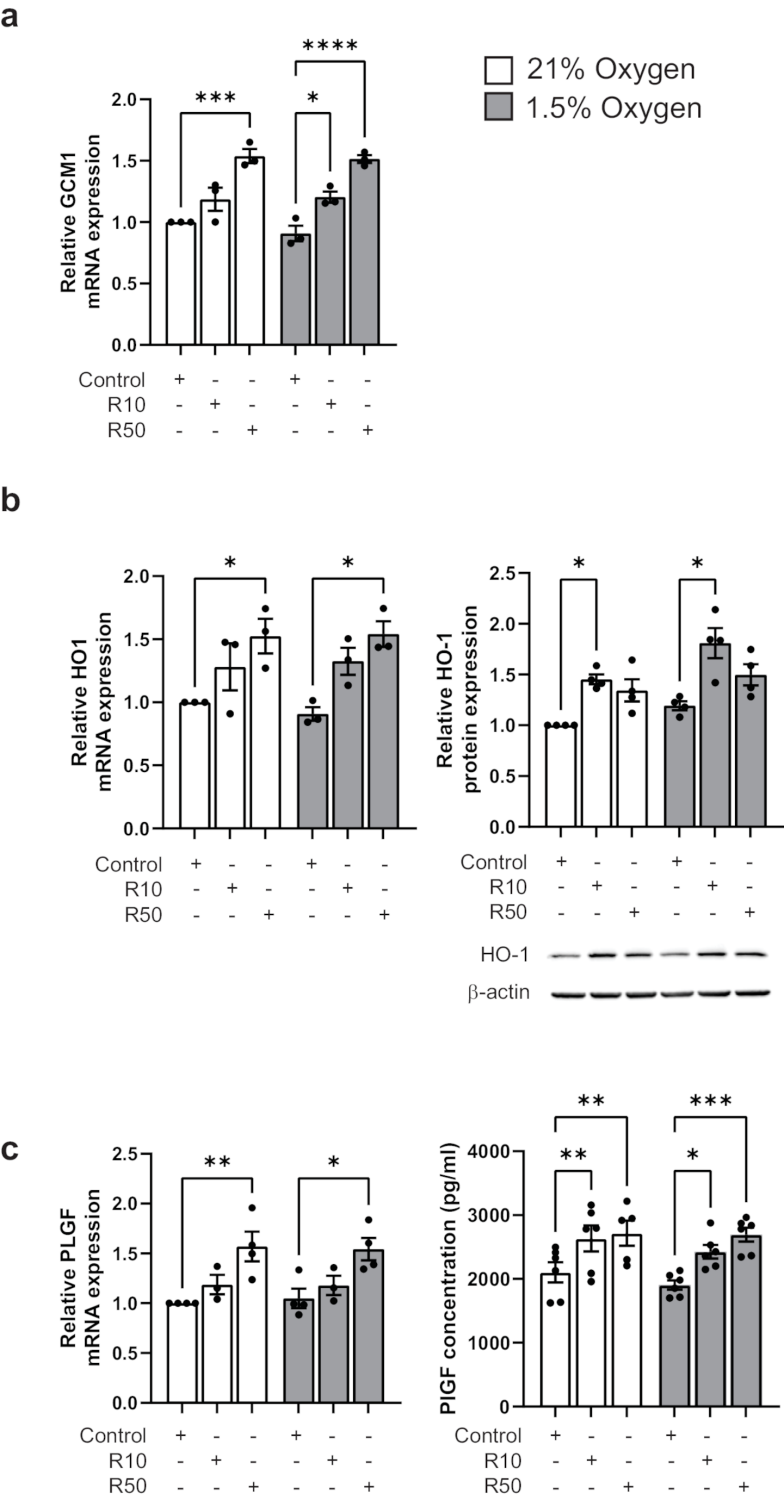
Rosiglitazone induces GCM1, HO-1 and PlGF expression. JEG-3 cells exposed to 21% O_2_ or 1.5% O_2_ (with reoxygenation at 21% O_2_) conditions in the presence of 10 (R10) or 50 (R50) µM rosiglitazone showed increased mRNA and protein expression of (**a**) GCM1, (**b**) HO-1, and (**c**) PlGF. Increases in protein were shown by western blot analysis (**b**) and increases in PlGF secretion by ELISA (**c**). All data are shown as mean ± SEM (*n* ≥ 3) and analysed by one-way ANOVA with post hoc tests; *P ≤ 0.05, **P ≤ 0.01, ***P ≤ 0.001, ****P ≤ 0.0001

PPARγ downstream target GCM1 mRNA was significantly induced with 50 µM rosiglitazone under 21% oxygen conditions (R50: 1.54 ± 0.06 vs. control: 1.0) and with both 10 and 50 µM doses under 1.5% oxygen/reoxygenation (R10: 1.21 ± 0.05, R50: 1.51 ± 0.03 vs. control: 0.91 ± 0.06) conditions (Fig. 2a). Another PPARγ target, HO-1, was induced with rosiglitazone treatment under 21% oxygen conditions (R50: 1.53 ± 0.14 vs. control: 1.0) and 1.5% oxygen/reoxygenation (R50: 1.54 ± 0.10 vs. control: 0.91 ± 0.05) conditions (Fig. 2b). Western blot analysis revealed rosiglitazone-mediated induction of HO-1 at the protein level under both 21% oxygen (R10: 1.45 ± 0.05 vs. control: 1.0) and 1.5% oxygen/reoxygenation (R10: 1.81 ± 0.15 vs. control: 1.19 ± 0.04) conditions (Fig. 2b). Activation of PPARγ signaling with rosiglitazone (50 µM) resulted in significant induction of PlGF mRNA expression under 21% oxygen (R50: 1.57 ± 0.15 vs. control: 1.0) and under 1.5% oxygen/reoxygenation (R50: 1.54 ± 0.11 vs. control: 1.05 ± 0.10) conditions (Fig. 2c). Rosiglitazone treatment also resulted in significant increases in PlGF protein secretion under 21% oxygen (R10: 2636 ± 203 pg/ml, R50: 2719 ± 197 pg/ml vs. control: 2105 ± 159 pg/ml) and 1.5% oxygen/reoxygenation (R10: 2425 ± 108 pg/ml, R50: 2694 ± 110 pg/ml vs. control: 1906 ± 73 pg/ml) conditions (Fig. 2c). This data suggests the involvement of PPARγ signaling in PlGF regulation.

### T0070907-Mediated Inhibition of PPARγ Signaling Reduced Basal Expression of PlGF which was Rescued by Rosiglitazone

**Fig. 3:**
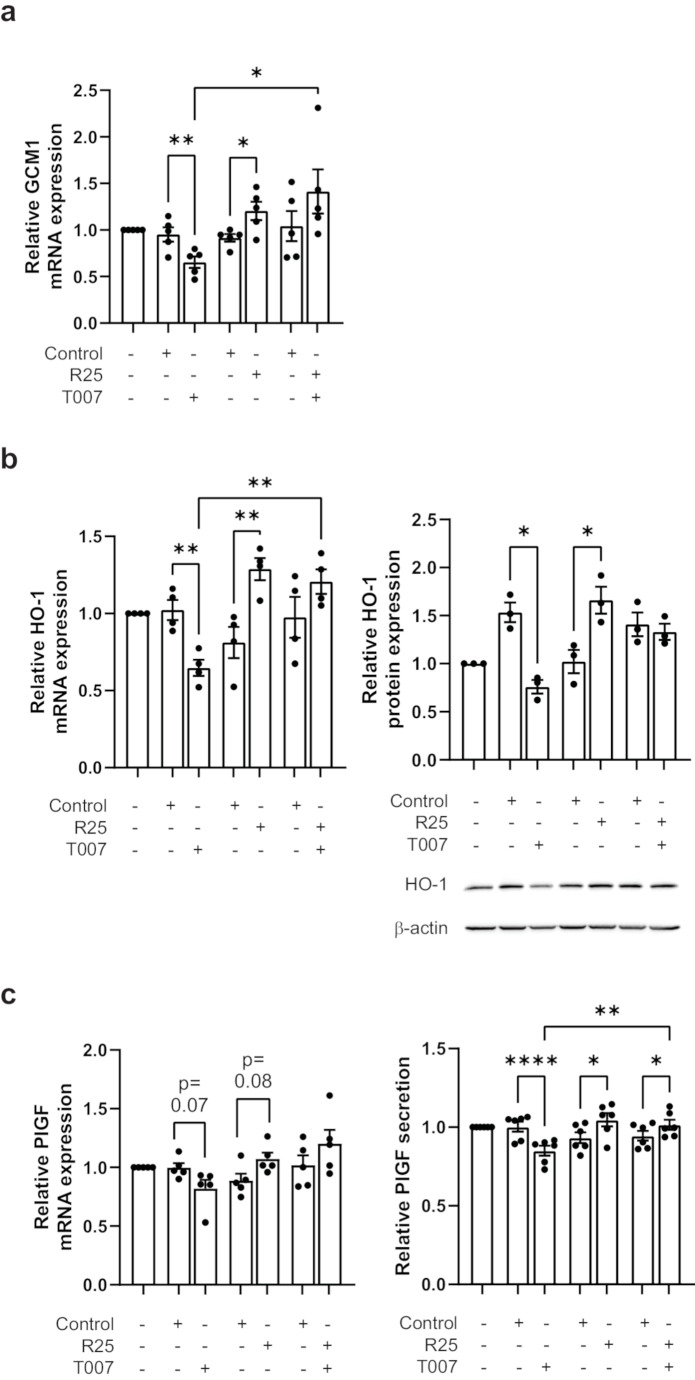
T0070907-mediated inhibition of basal GCM1, HO-1 and PlGF expression is rescued by rosiglitazone. JEG-3 cells were treated with vehicle control, T0070907 (T007, 1 µM), rosiglitazone (R25, 25 µM) or both T007 and R25 for 24 h exposed to 21% O_2_. mRNA expression was quantified for (**a**) GCM1, (**b**) HO-1 and (**c**) PlGF by RT-qPCR. Protein levels were quantified by western blot analyses for (**b**) HO-1 and secreted PlGF protein was determined by ELISA (**c**). All data are shown as mean ± SEM (*n* = 3) and analysed by one-way ANOVA with post hoc tests; *P ≤ 0.05, **P ≤ 0.01, ****P ≤ 0.0001

To further elucidate the involvement of PPARγ signaling in PlGF regulation, PPARγ was inhibited by treating JEG-3 cells with antagonist T007. Three different doses of T007 (1 µM, 5 µM and 10 µM) and two different time-points (6 h and 24 h) were tested to select the optimum treatment condition. As such, cells were treated with either 1 µM T007, 25 µM rosiglitazone or both for 24 h. The mRNA levels of both GCM1 (T007: 0.65 ± 0.06 vs. control: 0.95 ± 0.08) and HO-1 (T007: 0.65 ± 0.05 vs. control: 1.023 ± 0.07) were significantly reduced compared to the vehicle control with 1 µM T007 treatment for 24 h (Fig. 3a and b). Western blot analysis showed T007 reduced expression at the protein level for HO-1 (Fig. 3b; T007: 0.76 ± 0.07 vs. control: 1.535 ± 0.10). This data confirmed the inhibition of PPARγ signaling with T007 treatment. Rosiglitazone treatment alone or in the presence of T007 showed significant induction of GCM1 (rosiglitazone: 1.21 ± 0.10 vs. control: 0.92 ± 0.04, rosiglitazone + T007: 1.41 ± 0.24 vs. control: 1.04 ± 0.16) and HO1 (rosiglitazone: 1.29 ± 0.07 vs. control: 0.81 ± 0.10, rosiglitazone + T007: 1.21 ± 0.08 vs. control: 0.98 ± 0.13) mRNA compared to respective vehicle controls (Fig. 3a and b). Western blot analysis also revealed significant increase in HO-1 at the protein level with rosiglitazone treatment (Fig. 3b; rosiglitazone: 1.67 ± 0.14 vs. control: 1.02 ± 0.12). Inhibition of PPARγ signaling resulted only in a trend of reduced PlGF mRNA expression. However, a significant decrease in PlGF secretion by inhibition of PPARγ signaling by T007 was rescued with rosiglitazone (Fig. 3c; T007: 0.73 ± 0.03 vs. control: 1.00 ± 0.03, rosiglitazone: 1.05 ± 0.04 vs. control: 0.93 ± 0.03, T007 + rosiglitazone: 1.02 ± 0.03 vs. control: 0.95 ± 0.03). This data suggests that PPARγ signaling plays a significant role in PlGF regulation in trophoblast cells.

### PPARγ and GCM1 Silencing Reduced Basal Level PlGF Expression in Trophoblast Cells

**Fig. 4:**
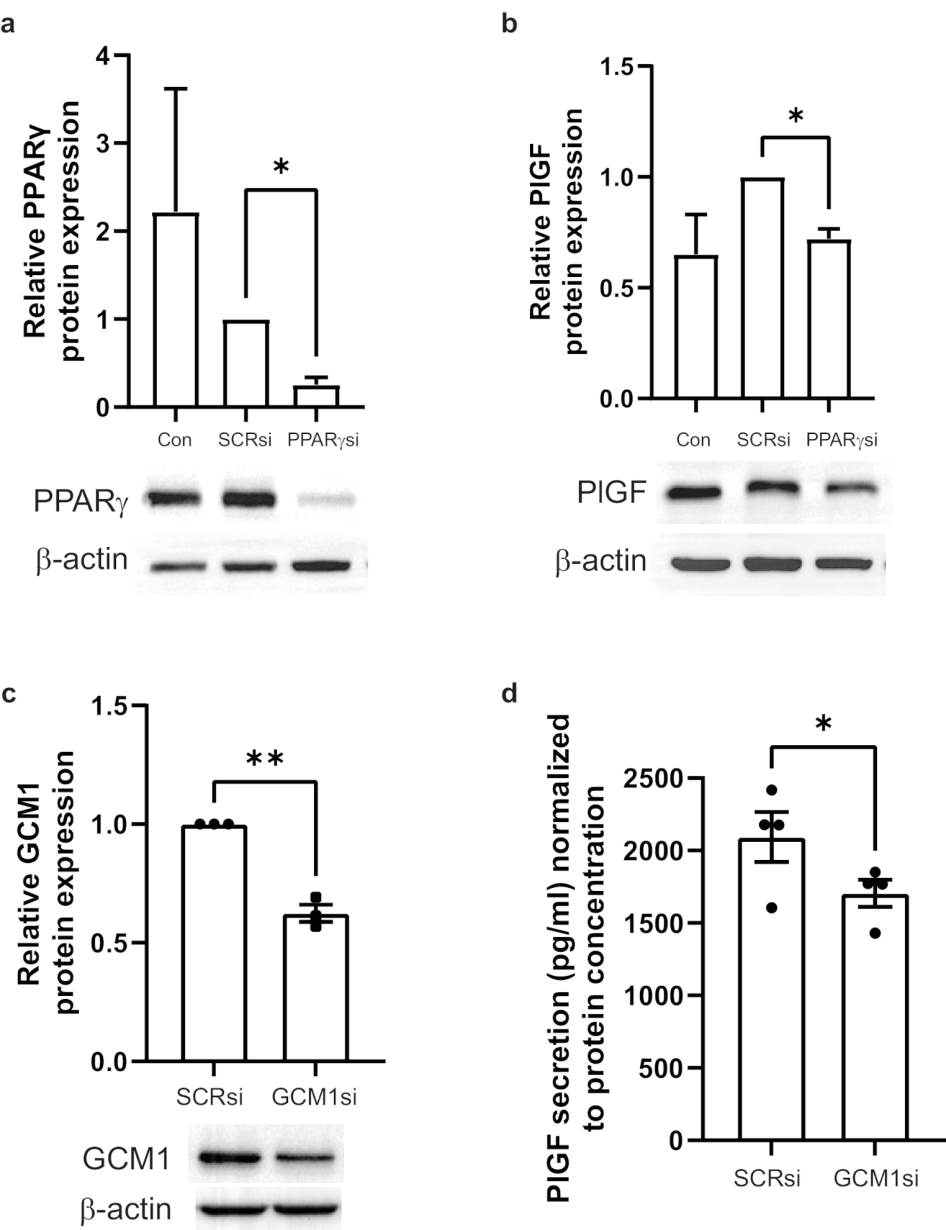
PPARγ knockdown reduces basal levels of PlGF expression. JEG-3 cells were transduced with PPARγ siRNA (PPARγsi, 50 pmol) or scrambled siRNA (SCRsi, 50 pmol) and lysed for western blot analyses for (**a**) PPARγ or (**b**) PlGF. JEG-3 cells were transduced with GCM1 siRNA (GCM1si, 75 pmol) or scrambled siRNA (75 pmol) and (**c**) lysed for western blot analysis for GCM1 or (**d**) PlGF secretion levels determined by ELISA. All data are shown as mean ± SEM (*n* = 3) and analysed by paired t-test; *P ≤ 0.05, **P ≤ 0.01

To confirm the role of PPARγ-GCM1 axis in PlGF regulation, PPARγ or GCM1 was silenced in JEG-3 cells using siRNAs. Knockdown was confirmed with western blot analysis which showed approximately 75% reduced expression for PPARγ and 38% decreased expression of GCM1 respectively compared to an siRNA negative control (Fig. 4a and c; PPARsi: 0.26 ± 0.08 vs. SCRsi: 1.00 ± 0.00, GCM1si: 0.62 ± 0.04 vs. SCRsi 1.00 ± 0.00). The effect of PPARγ knockdown on PlGF expression was assessed with western blotting which revealed significant reduction in PlGF basal level expression (Fig. 4b; PPARsi: 0.72 ± 0.04 vs. SCRsi: 1.00 ± 0.00). The effect of GCM1 knockdown on PlGF expression was confirmed with ELISA which showed small but significant decrease in PlGF basal expression (Fig. 4d; GCM1si: 1705 ± 94 vs. SCRsi: 2094 ± 173). These data confirmed the importance of PPARγ-GCM1 axis signaling in PlGF regulation in trophoblast cells.

## Discussion

This study aimed to elucidate the molecular links between PPARγ-GCM1-mediated trophoblast differentiation and pro-angiogenic PlGF expression and its potential for pharmacological modulation.

Using a JEG-3 choriocarcinoma trophoblast cell model under 21% oxygen and 1.5% oxygen/reoxygenation conditions, our study explored the effect of PPARγ activation on PlGF expression and secretion in a setting relevant to the low oxygen stress observed in PE. PPARγ activity was manipulated through pharmacological intervention via its agonist, rosiglitazone, or its antagonist, T0070907, to measure its effect on PlGF. Lastly, we observed that changes in PlGF levels and secretion were at least in part regulated by PPARγ and GCM1 linking for the first time trophoblast differentiation with PlGF expression.

To evaluate the potential effects of rosiglitazone-mediated activation of PPARγ on PlGF, we treated cells with physiological concentrations (10–50 µM) of the drug. Following rosiglitazone treatment, both PlGF mRNA expression and protein secretion were significantly increased under all culture conditions. Known downstream targets of PPARγ, such as the trophoblast differentiation marker and transcription factor GCM1 and the cytoprotective HO-1 were both positively regulated by rosiglitazone exposure. Consistent with previous research indicating that PPARγ activation can lead to receptor degradation, we also noted a decrease in PPARγ expression in response to rosiglitazone treatment in our JEG-3 model [24, 25].

**Fig. 5:**
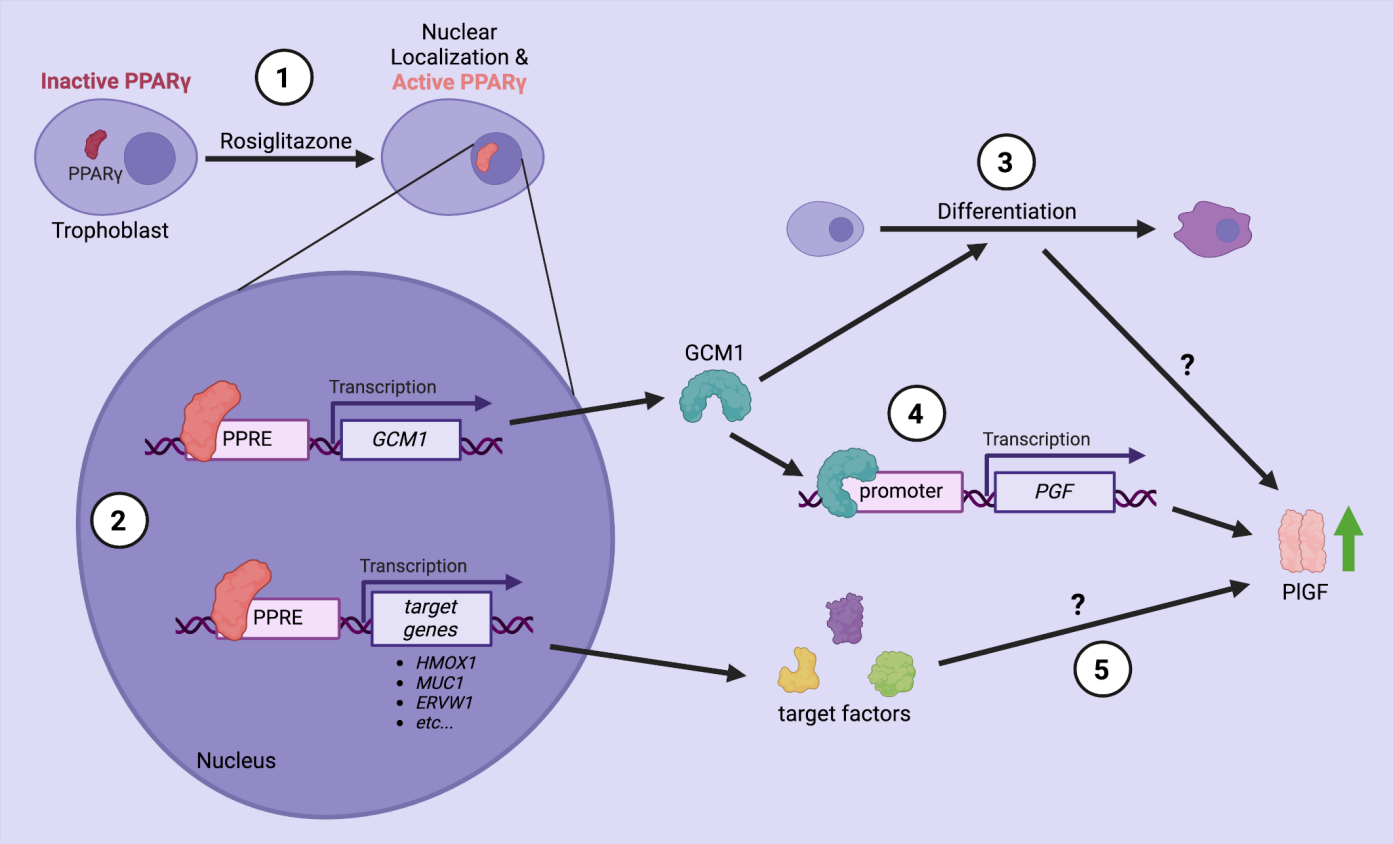
PPARγ-mediated PlGF regulation in a trophoblast cell line. Placental pathology is often characterised by abnormal trophoblast differentiation, oxidative stress, inflammation and reduced expression of PPARγ, GCM1 and PlGF. In this study, we explored whether activation of PPARγ using rosiglitazone could restore PlGF expression and secretion under 21% O_2_ and 1.5% O_2_ conditions. Our finding suggests that rosiglitazone (1) induces nuclear translocation of PPARγ; (2) promotes transcription of GCM1, HO1, and other PPARγ target genes; (3) might support trophoblast differentiation via GCM1 as shown previously [30]; (4) regulates PLGF expression in part via GCM1, as siRNA-mediated knockdown of GCM1 led to reduced PlGF secretion in our trophoblast model, aligning with previous finding [17]. (5) Some other yet unknown factors might also contribute to the regulation of PlGF expression. Created with BioRender.com

To ensure that these effects were PPARγ specific, we used two approaches. First, we inhibited PPARγ activity using T0070907, a specific antagonist that functions by altering the conformation of the PPARγ ligand-binding domain and its interaction with co-factors [26]. Second, we used siRNA interference to reduce PPARγ expression in our model. Both approaches showed that inhibition of PPARγ expression and activity resulted in significant reduction in PlGF protein expression/secretion. Our data provide compelling evidence that pharmacological modulation of PPARγ activity regulates PlGF expression. While rosiglitazone treatment resulted in significant increases in GCM1 mRNA expression, we observed a trend of increased GCM1 protein levels that was not significant (data not shown). Other studies have identified increased GCM1 protein levels in response to PPARγ activation [22] and GCM1 transcriptional regulation of PlGF [18]. This suggests there may be other pathways downstream of PPARγ involved in PlGF expression/secretion that have yet to be identified (Fig. 5). Nonetheless, the nature of this regulation, whether direct or indirect, remains to be fully elucidated.

Utilizing murine trophoblast stem cell model, Parast et al. showed that PPARγ drives trophoblast differentiation by the activation of GCM1, the transcription factor critical for trophoblast differentiation and syncytial fusion [27]. We have previously demonstrated in human trophoblast cell models that PPARγ is able to induce cell differentiation via the activation of GCM1 [28]. In the present study we observed an increase in PlGF expression with PPARγ activation, suggesting an interrelated signaling pathway involving PPARγ, GCM1, and PlGF that may facilitate placental angiogenesis and function. These findings align with prior research demonstrating the regulation of PlGF by GCM1 in the trophoblast [17], reinforcing the pivotal role of PPARγ as a transcriptional regulator in placentation and its potential as a therapeutic target in PE.

The JEG-3 choriocarcinoma cell line provided a controlled in vitro environment to simulate low oxygen/reoxygenation injury characteristic of PE. While the JEG-3 model is well-established for studying trophoblast function, and our findings using this model recapitulate the observations made in various other cell and tissue-based models, it may not fully replicate the in vivo complexity of placental development. Future studies should aim to validate these findings in primary trophoblast cultures and animal models to confirm their translational potential.

Additionally, our results demonstrate a dose-dependent increase in PlGF expression with rosiglitazone treatment. Given the known cardiovascular risks associated with high-dose rosiglitazone, such as myocardial infarction, it is crucial to balance these potential adverse effects with the therapeutic benefits of PPARγ activation [29]. Careful dose optimization will be essential for future clinical applications. Furthermore, our inhibition studies highlight the critical role of PPARγ in regulating PlGF expression. While the off-target effects of pharmacological inhibitors and siRNA were considered in the design of this study, future work with genetic knockout mouse models should be conducted to ensure the specificity of PPARγ-mediated regulation of its potential downstream effectors, including PlGF.

Despite providing valuable insights, this study has several limitations. The in vitro nature of the experiments limits their direct applicability to clinical settings. Moreover, the long-term effects of PPARγ activation on placental and fetal development remain unknown. Future research should focus on longitudinal studies in animal models and clinical trials to evaluate the long-term safety and efficacy of PPARγ agonists. Exploring combination therapies with other angiogenic modulators may enhance therapeutic outcomes.

## Conclusion

In conclusion, our findings highlight the therapeutic potential of rosiglitazone-mediated PPARγ activation in upregulating PlGF and ameliorating placental dysfunction in PE, addressing a significant unmet need in maternal-fetal medicine. By targeting the PPARγ-GCM1-PlGF axis, this approach offers a promising avenue for developing effective treatments for PE.

## Electronic Supplementary Material

Below is the link to the electronic supplementary material.

Supplementary Material 1
