# Supplementary figures and images for: Rosiglitazone-Mediated Activation of PPARγ Induces PlGF Expression in Trophoblast Cells
Source: Reprod Sci. 2025 Apr 28;32(6):1864–75. doi: 10.1007/s43032-025-01868-w (PMC12187888; doi:10.1007/s43032-025-01868-w)

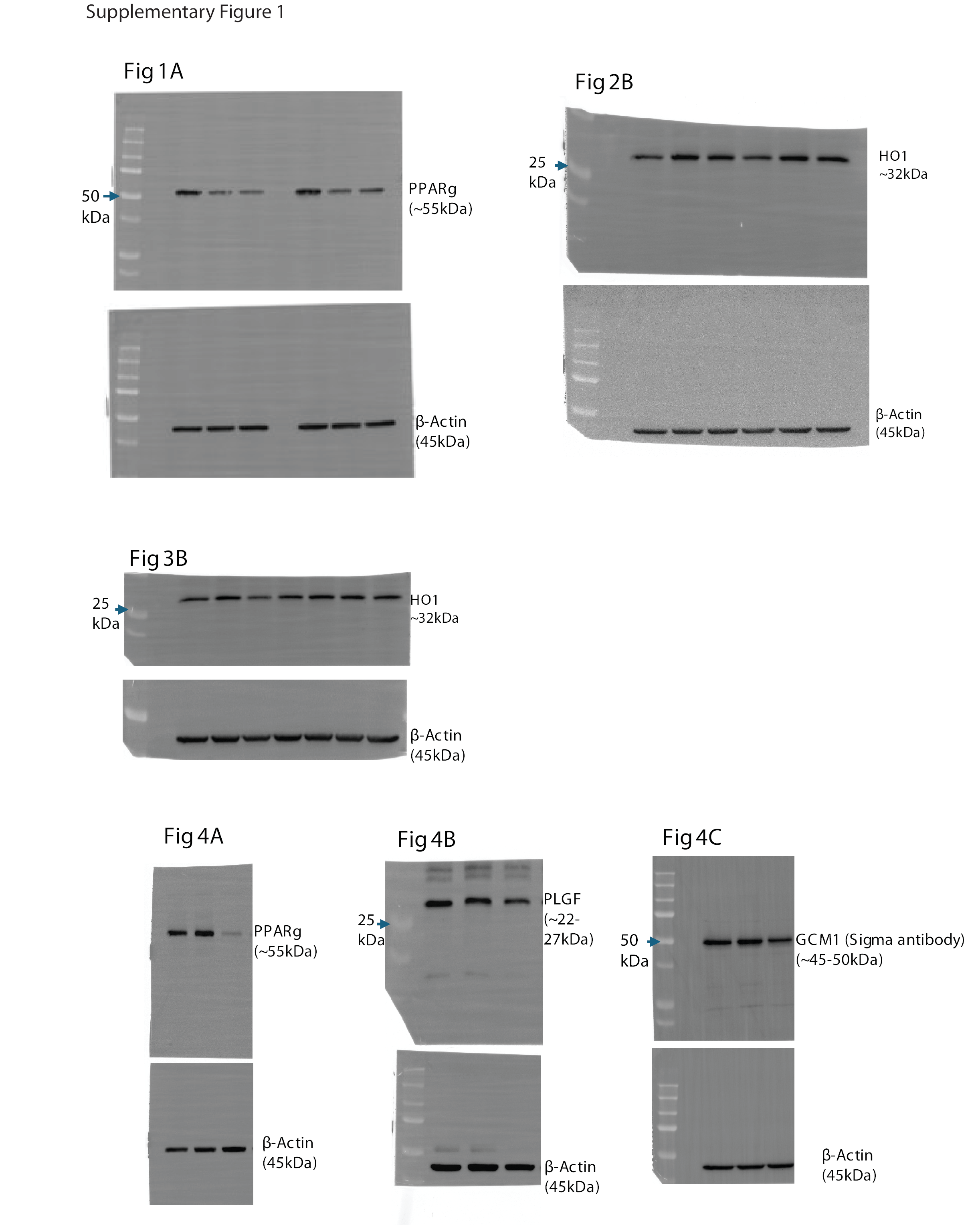

Supplement: Supplementary file 1 — Supplementary Material 1 [file 43032_2025_1868_MOESM1_ESM.png]
